# Sparse polynomial surrogates for F-actin networks with compliant crosslinkers

**DOI:** 10.1007/s10237-026-02067-5

**Published:** 2026-06-03

**Authors:** Luís Pacheco, Marco Parente, João Ferreira

**Affiliations:** 1 Department of Mechanical Engineering, Faculty of Engineering, University of Porto, R. Dr. Roberto Frias, 4200-465 Porto, Portugal; 2 Institute of Mechanical Engineering and Industrial Management, R. Dr. Roberto Frias, 4200-465 Porto, Portugal

**Keywords:** Actin networks, Continuum mechanics, Finite elements, Sparse polynomial chaos expansions, Uncertainty quantification

## Abstract

Filamentous actin (F-actin) constitutes the primary contributor to cell elasticity and structural integrity, forming dynamic, crosslinked networks in the actin cortex. Existing mechanical models for F-actin and crosslinked filament networks successfully describe filament- and network-level behavior, but are often limited in accounting for biological dynamic processes and inherent material uncertainty and variability. We develop a stochastic modeling framework that integrates Polynomial Chaos Expansion (PCE) surrogates using the Finite Element Method (FEM). These surrogates replace filament-scale equations for compliant crosslinked F-actin networks, efficiently enabling uncertainty quantification and sensitivity analysis of key material parameters. The first and second statistical moments from the PCE are incorporated into a micro-sphere network model and implemented via a user-defined material subroutine. Validation was performed against 10 000 Monte Carlo simulations (MCS) for each of four FEM test cases: three simple deformation modes applied to a unit length cubic element, and a thin gel layer under shear mimicking a parallel plate rheology setup. In every test, the surrogate predicts the expected value of relevant stress quantities at maximum deformation with under 1% relative error versus the MCS reference. Moreover, the surrogate captures the network’s variability as measured by second-order moments, demonstrating its ability to deliver rapid, statistically faithful predictions of both mean response and standard deviation in simple element tests and experimentally relevant rheology geometries. The proposed methodology provides a scalable route for incorporating intrinsic material variability into F-actin mechanical modeling, with implications for studying cell motility, division, and pathologies related to cytoskeletal remodeling.

## Introduction

Cells actively sense and generate forces by adjusting their mechanical properties through mechanosensing and mechanotransduction processes. These functions are driven by the cytoskeleton, which is the main component responsible for cell shape regulation and mechanical support. The cytoskeleton is a composite material made of three types of filamentous polymers - microtubules, intermediate filaments, and actin filaments (F-actin) - along with regulatory proteins that control filament formation, growth and disassembly (Fletcher and Mullins 2010; Burla et al. 2019).

F-actin occupies almost twice as much volume as the remaining filaments, thus establishing it as a crucial driver of cell elasticity and structural function (Kelkar et al. 2020; Haspinger et al. 2021). These semiflexible polymers are dynamically organized into complex higher-order networks through the coordinated action of active motor and passive crosslinker proteins (Blanchoin et al. 2014). Such networks form the structural basis of the actin cortex, an active cellular component located beneath and tethered to the plasma membrane. Crosslinker proteins, or simply crosslinkers, bind and unbind to F-actin in continuous cycles. Therefore, the mechanics of the actin cortex depend strongly on their binding-unbinding rates and relative concentrations, with some crosslinkers being rigid (e.g., scruin) and others compliant (e.g., filamin, $\alpha$-actinin) (Shin et al. 2004). Understanding cytoskeletal mechanics, and the actin cortex in particular, is crucial as it influences key cellular processes such as cell motility, division, and differentiation (Pollard and Cooper 2009; Pegoraro et al. 2017). Moreover, remodeling of the actin cytoskeleton has emerged as a significant biomarker in carcinogenesis and enhanced malignancy (Suresh 2007; Hosseini et al. 2021; Suresh and Diaz 2021). Consequently, mechanical models have been developed to describe the behavior of F-actin and networks of crosslinked filaments.

At the filament scale, the key objective is to obtain a force-extension relationship. F-actin is commonly modeled as a worm-like chain, in which bending stiffness is determined by the persistence length. This description originates from the theoretical framework of Kratky and Porod (1949), with the first numerical implementation provided by Fixman and Kovac (1973). While statistical mechanics approaches are common, fully mechanical models have also been developed. Continuum mechanics formulations for both inextensible and extensible biopolymer filaments were presented by Holzapfel and Ogden (2011), addressing inconsistencies between physical and mechanical descriptions. This framework was later generalized (Unterberger et al. 2013a; Holzapfel and Ogden 2013) through the introduction of an exponent parameter (the $\beta$-model), thereby reconciling the mechanical formulation with physics-based models such as those in MacKintosh et al. (1995) and Blundell and Terentjev (2009). The work of Unterberger et al. (2013a) further applied homogenization at the network scale by utilizing the non-affine micro-sphere model introduced by Miehe et al. (2004). Viscoelasticity was introduced in such networks by Unterberger et al. (2013b), while Holzapfel et al. (2014) demonstrated that modeling deformable crosslinker proteins within an affine full-network model effectively captures the shear and normal stress responses of semiflexible biopolymer networks. The active role of myosin motors in contractile bundles was incorporated by Ferreira et al. (2018). Additionally, the $\beta$-model, embedded in the cytosol, has been applied to multiscale Finite Element Method (FEM) simulations in Klinge et al. (2018).

While effective in capturing the behavior of F-actin at both filament and network levels, mechanical models still face important limitations. They are often characterized by high complexity and a wide parameter space, with several parameters lacking direct physical interpretation. Moreover, their deterministic nature restricts the analysis to the general characteristics of a system, making them unsuitable for a reliable study of systems with inherent uncertainties in boundary and loading conditions, as well as geometric and material properties. Dynamic cytoskeletal processes such as filament (de)polymerization, severing, and crosslinker binding, coupled with a vast diversity of crosslinker proteins, result in microstructural heterogeneity and significant material variability. Hence, a deterministic approach based on a single value for each filament and crosslinker property fails to capture the true complexity of F-actin networks. Furthermore, parameter estimation relies on experimental data, which is often scarce, being itself subject to measurement noise and variability. As highlighted by Famaey et al. (2025), significant discrepancies were observed among laboratories analyzing biological samples under simple experimental conditions, despite the use of a unified protocol.

Some of these limitations can be addressed through the use of stochastic methods, which are able to capture material variability and provide better model insights through efficient sensitivity analysis. To account for the stochastic nature of a system, random fields are incorporated into deterministic FEM, leading to the Stochastic FEM (SFEM). There are several variants of the SFEM, from the classical Monte Carlo simulations (MCS) to more computationally robust techniques, such as the Perturbation Method (PM) (Liu et al. 1986; Kleiber and Hien 1992) and the Spectral SFEM (SSFEM), based on Polynomial Chaos Expansion (PCE) (Ghanem and Spanos 1990; Anders and Hori 2001). MCS is a non-intrusive method that requires numerous simulations to obtain accurate results. In contrast, both the PM and SSFEM are intrusive approaches. PM relies on approximating the response vector using a Taylor series expansion, whereas SSFEM represents uncertain input parameters through Karhunen-Loève expansions and characterizes the probabilistic behavior of the system response via PCE. Despite their potential advantages, the implementation of intrusive methods can be challenging, particularly in complex FEM simulations involving highly nonlinear hyperelastic materials or the network applications herein discussed.

A widely adopted, non-intrusive technique is the independent use of PCE for surrogate model construction (Berveiller et al. 2006; Sudret 2008), providing a computationally efficient means of obtaining the model’s quantities of interest, while serving as a powerful tool for uncertainty quantification.

PCE surrogates have proven to be a valuable tool in biological contexts, particularly for handling inherent material variability. These models are commonly employed to replace FEM formulations in parameter estimation procedures. For instance, in cardiac mechanics, they have been successfully used to replace the Holzapfel-Ogden model within a FEM implementation (Campos et al. 2023). Further applications include estimating pressure and dynamic corneal response (Yaghoubi et al. 2022), performing uncertainty network analysis of cardiac mechanosignaling (Cao et al. 2020), and quantifying and propagating uncertainty in patient-specific arterial elasticity predictions (Roy et al. 2025).

Despite their widespread use, PCE surrogates are not ideally suited for fully replacing FEM F-actin network models, as these often require varying boundary conditions or adapting the network to different geometries. In this work, PCE surrogates are leveraged to replace the filament-level equations presented in Holzapfel et al. (2014), enabling uncertainty quantification and sensitivity analysis of input parameters related to the material properties of both F-actin and compliant crosslinkers. The first- and second-order statistical moments obtained from the surrogate polynomial are incorporated into the micro-sphere model (Miehe et al. 2004), which is then implemented within a user-defined material subroutine (UMAT) in commercial FEM software Abaqus (Dassault Systèmes, France). This approach allows capturing the full range of the network’s mechanical behavior arising from material parameter variability, without requiring a large number of simulations or modifying the original equilibrium equations.

Section 2 outlines the methodology employed in this study. We first describe the key relationships governing filament mechanics, highlighting those incorporated into the PCE surrogate models to represent the quantities of interest. The homogenization process within an affine network framework is then presented, followed by the details of the implementation within a FEM context and representative numerical examples. A concise overview of PCE is provided, including a description of the input parameters, their probabilistic distributions, the procedures for calculating PCE coefficients, and the approach adopted for sensitivity analysis. Section 3 presents and discusses the results. The filament surrogates are validated against the original mechanical equations, uncertainty quantification is performed for the quantities of interest, and Sobol-based sensitivity analysis is conducted to identify the most influential parameters. Additionally, FEM simulations utilizing the PCE surrogates are compared to MCS for both simple deformation modes and a parallel-plate rheology setup. Finally, Sect. 4 presents the main conclusions of this work and the perspectives for future research.

## Methods

This section describes the mechanical relationships governing crosslinked F-actin filaments, together with the kinematic principles and constitutive relations used for their homogenization into an affine network. It then introduces the PCE framework, including the characterization of input parameters and the post-processing of results for sensitivity analysis and uncertainty quantification. PCEs were implemented using the *chaospy* library (Feinberg and Langtangen 2015). All continuum mechanics equations referenced here can be found in established textbooks such as Holzapfel et al. (2000), and further details on UMAT implementation are available in Abaqus documentation (Smith 2009), with several implementation examples available at https://github.com/jpsferreira/UMAT-ABAQUS_library.

### F-actin with compliant crosslinkers

**Fig. 1:**
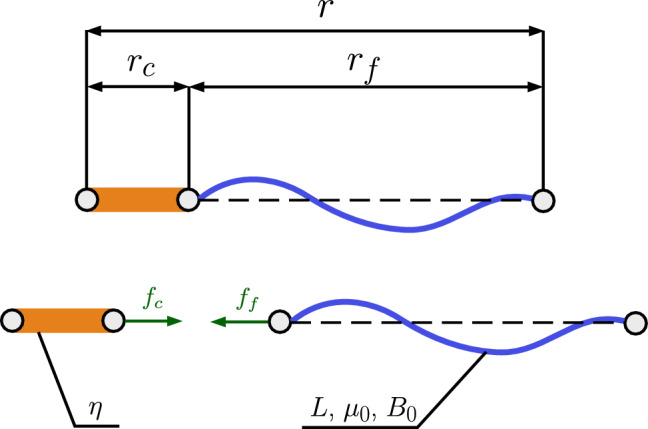
Schematic of the series mechanical model for a crosslinker (orange bar) and an actin filament (wavy blue line). The compound filament end-to-end distance *r* is defined by the sum of the crosslinker and actin filament end-to-end distances, $r_c \sim \mathcal {O}(10^1) \, \text {nm}$ and $r_f \sim \mathcal {O}(10^0) \, \mu \text {m}$, respectively. The lower panel provides the free-body diagram depicting internal equilibrium forces $f_c$ and $f_f$. The mechanical response is governed by the crosslinker relative stiffness $\eta$ and the actin filament contour length $L \sim \mathcal {O}(10^1) \, \mu \text {m}$, stretch modulus $\mu _0$ and bending stiffness $B_0$

The continuum mechanical model of crosslinked F-actin with compliant linkers (Holzapfel et al. 2014) considers a compound filament structure, where each actin filament is connected to a flexible crosslinker protein, as illustrated in Fig. 1. The crosslinker can only carry loads in the axial direction. In the absence of external forces, the filament and linker have end-to-end distances $r_{0,f}$ and $r_{0,c}$, respectively, making a total end-to-end distance of the compound $r_0 = r_{0,f} + r_{0,c}$. After deformation, the end-to-end distances become $r_f$ and $r_c$, resulting in a total end-to-end distance of $r = r_f + r_c$.

For the actin filament and crosslinker, the stretches are defined as $\lambda _f = r_f/r_{0,f}$ and $\lambda _c = r_c/r_{0,c}$, respectively. Hence, the total stretch of the compound is given by:

1 $$\begin{aligned} \lambda r_0 = \lambda _f r_{0,f} + \lambda _c r_{0,c} ~\mathrm {.} \end{aligned}$$

As depicted in the free-body diagram in Fig. 1, the force acting on the filament must be equal to the force acting on the crosslinker, such that $f_f = f_c = f$. The elongation of the compound $\Delta r$ can also be split into $\Delta r = \Delta r_f + \Delta r_c$. A material parameter is introduced to define the ratio between the elongation of the compound ($\Delta r$) and that of the actin filament ($\Delta r_f$). It is referred to as the relative stiffness of the crosslinker, $\eta \in (0,1]$:

2 $$\begin{aligned} \eta = \frac{\Delta r_f}{\Delta r} ~{.} \end{aligned}$$

The limit case $\eta = 1$ corresponds to a rigid crosslinker, where the stretch applied to the compound is entirely absorbed by the actin filament, while $\eta = 0$ corresponds to the absence of a crosslinker protein. The filament stretch $\lambda _f$ can then be expressed as a function of the total stretch $\lambda$ and relative stiffness $\eta$:

3 $$\begin{aligned} \lambda _f = \eta \frac{r_0}{r_{0,f}}(\lambda - 1) + 1 ~{,} \end{aligned}$$

and the crosslinker stretch $\lambda _c$ can be obtained from Eq. (1).

In order to embed the compound filament model in a network framework, one can assume that the compound is pre-stretched, by adding the network parameter $\lambda _0$, which relates to the initial end-to-end distance in the unloaded network $\tilde{r}$ by means of $\lambda _0 = \tilde{r}/r_0$. By doing so, the network admits a prestressed state, capable of replicating *in vivo* conditions. The mechanical response of the filaments is captured by the force-stretch relation described in Unterberger et al. (2013a):

4 $$\begin{aligned} \begin{aligned}&\frac{\lambda _f \lambda _{0,f} r_{0,f}}{L}= \\&=1+\frac{f}{\mu _0} + \frac{(1+2f/\mu _0)(1+f/\mu _0)^\beta (1-r_{0,f} / L)}{[1+fL^2/(\pi ^2B_0)+f^2L^2/(\pi ^2B_0\mu _0)]^\beta } {,} \end{aligned} \end{aligned}$$

where *L* represents the filament contour length, $\mu _0$ is the stretch modulus, $B_0$ denotes the bending stiffness, and $\beta$ is a dimensionless parameter. Bending stiffness is calculated as $B_0 = T L_p k_B$, where *T* is the temperature, $L_p$ is the persistence length and $k_B$ is the Boltzmann constant. Filament force *f* is evaluated implicitly for a given stretch $\lambda _f$ using derivative-free Brent’s method (Brent 1971). The first derivative of the free energy $w^\prime$ is obtained by applying the chain rule to $f = \partial w/\partial r$, leading to:

5 $$\begin{aligned} w^\prime = \partial w/\partial \lambda = f \lambda _{0} r_{0} ~{.} \end{aligned}$$

The second derivative of the free energy $w^{\prime \prime }$ is given by implicit differentiation, after rearranging Eq. (4) into the form $H[\lambda _f(\lambda ), w^\prime (\lambda )] = 0$:

6 $$\begin{aligned} \frac{dH}{d\lambda } = \frac{\partial H}{\partial \lambda _f} \frac{\partial \lambda _f}{\partial \lambda } + \frac{\partial H}{\partial w^\prime } \frac{\partial w^\prime }{\partial \lambda } = 0 ~{,} \end{aligned}$$

and replacing the definition of $w^{\prime \prime } = \partial w^\prime / \partial \lambda$, it follows that

7 $$\begin{aligned} w^{\prime \prime } = -\frac{\partial H / \partial \lambda _f}{\partial H / \partial w^\prime } \frac{\partial \lambda _f}{\partial \lambda } ~{.} \end{aligned}$$

After some algebraic manipulation, and by introducing the dimensionless parameter $\alpha = \pi ^2 B_0 / (\mu _0 L^2)$, the final expression for $w^{\prime \prime }$ reads:

8 $$\begin{aligned} w^{\prime \prime }&= \frac{\eta \lambda _{0,f} \lambda _{0} r_{0}^{2} \mu _{0} / L}{1+Y\left( \frac{1+\alpha f^{*}}{1+f^{*}+\alpha {f^{*}}^{2}}\right) ^{\beta }\left( 1-r_{0,f} / L\right) } {,} \end{aligned}$$

9 $$\begin{aligned} \text {with} \quad Y&= \frac{\beta }{\alpha } \frac{\left( 1+2 \alpha f^{*}\right) ^{2}}{1+f^{*}+\alpha {f^{*}}^{2}}-\beta \frac{1+2 \alpha f^{*}}{1+\alpha f^{*}}-2 {.} \end{aligned}$$

The dimensionless force $f^*$ is defined as:

10 $$\begin{aligned} f^* = f \frac{L^2}{\pi ^2 B_0} ~{.} \end{aligned}$$

**Fig. 2:**
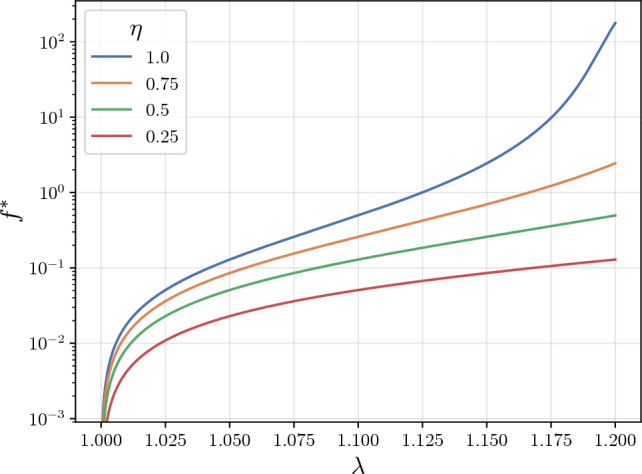
Influence of crosslinker relative stiffness $\eta$ on the force-stretch relation. The remaining material parameters are set to: $r_{0,c}=14\,\text {nm}$, $r_{0,f}=1.63 \, \mu \text {m}$, $L = 1.96 \, \mu \text {m}$, $\beta = 0.5$, $\mu _0 = 38.6 \, \text {nN}$, $L_p = 16 \, \mu \text {m}$ and $T = 294 \, \text {K}$

Figure 2 illustrates the effect of the relative stiffness $\eta$ on the compound’s force-stretch relation: varying the crosslinker stiffness alone shifts the force response and, for higher stretches, causes it to span several orders of magnitude. The surrogate models developed in this work and presented in Sect. 2.4 generate predictions for *f*, $w^\prime$ and $w^{\prime \prime }$, thereby strictly replacing Eqs. (4), (5) and (8) in the constitutive formulation. In contrast, the subsequent homogenization into an affine network follows the original constitutive formulation, being explicitly solved by numerical integration within the finite element routine.

### Homogenization into an affine network

#### Kinematics and strain-energy functions

Considering a continuum body $\mathcal {B}$ in three-dimensional Euclidean space as it moves in space through time instants, it occupies a continuous sequence of geometrical regions, defined as configurations $\Omega _0,..., \Omega$. The configuration $\Omega _0$ at time $t=0$ is the reference configuration, while the configuration of $\mathcal {B}$ at time *t*, denoted by $\Omega$, is the current configuration. A point in the reference configuration is represented by the position vector $\textbf{X}$, that maps to the associated point $\textbf{x}$ in the current configuration, through a continuous, differentiable and uniquely invertible motion vector field $\mathcal {X}$, such that $\textbf{x} = \mathcal {X}(\textbf{X}, t)$ and $\textbf{X} = \mathcal {X}^{-1}(\textbf{x}, t)$. Local deformation of the continuum body is described by the deformation gradient $\textbf{F}$:

11 $$\begin{aligned} \textbf{F}(\textbf{X}, t) = \frac{\partial \mathcal {X}(\textbf{X}, t)}{\partial \textbf{X}} ~{~.} \end{aligned}$$

The determinant of the deformation gradient $\textrm{det}\textbf{F} = J$ represents the volume ratio (Jacobian) associated with the deformation, and rotation-independent deformation is quantified by the right Cauchy-Green tensor $\textbf{C} = \textbf{F}^{\textrm{T}} \textbf{F}$. Incompressibility is enforced by adding a penalty parameter to the compressible formulation, requiring a multiplicative split of the deformation gradient $\textbf{F} = \textbf{F}_{\text {vol}} \bar{\textbf{F}}$, where $\textbf{F}_{\text {vol}} = J^{\frac{1}{3}} \textbf{I}$ is the volume-changing part and $\bar{\textbf{F}} = J^{-\frac{1}{3}} \textbf{F}$ is the isochoric component. Accordingly, the strain-energy function $\Psi$ is also decomposed as:

12 $$\begin{aligned} \Psi (\textbf{C}) = \Psi _{\text {vol}}(J) + \bar{\Psi }(\bar{\textbf{C}}) ~{~,} \end{aligned}$$

with $\Psi _{\text {vol}}(J)$ and $\bar{\Psi }(\bar{\textbf{C}})$ describing the volumetric and isochoric parts of the strain-energy function, respectively. The volumetric component can be written as proposed by Simo and Miehe (1992):

13 $$\begin{aligned} \Psi _{\textrm{vol}} = \frac{k}{4} \left( J^2 - 1 - 2\ln J \right) ~\textrm{,} \end{aligned}$$

where *k* is the bulk modulus. The isochoric part $\bar{\Psi }$ includes contributions from the affine filamentous network (AN) with a volume fraction $\phi$ and the hyperelastic isotropic matrix (IM):

14 $$\begin{aligned} \bar{\Psi }(\bar{\textbf{C}}) = \bar{\Psi }_{\textrm{AN}}(\bar{\textbf{C}}) + (1-\phi )\bar{\Psi }_{\textrm{IM}}(\bar{\textbf{C}}) ~{~.} \end{aligned}$$

**Fig. 3:**
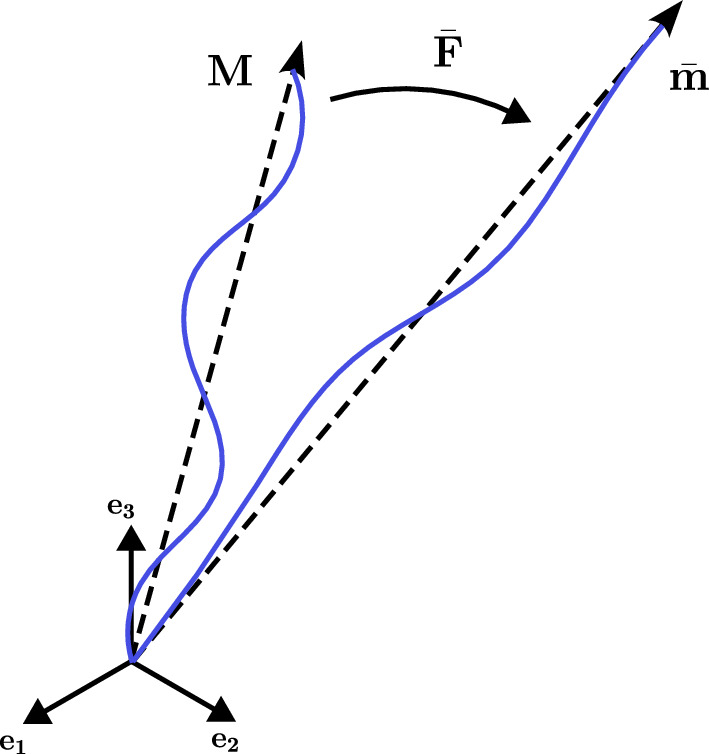
Filament mean orientation at reference and deformed configurations. The filament (wavy blue line) is initially oriented along unit vector $\textbf{M}$ (dashed arrow) in the reference configuration. Upon application of the isochoric deformation gradient $\bar{\textbf{F}}$, it is mapped to the deformed direction $\bar{\textbf{m}} = \bar{\textbf{F}} \cdot \textbf{M}$. The magnitude of $\bar{\textbf{m}}$ defines the isochoric stretch of the filament $\bar{\lambda } = \sqrt{\bar{\textbf{m}}\cdot \bar{\textbf{m}}}$

Here, $\bar{\Psi }_{\textrm{IM}}$ represents the mechanical response of the medium in which the filaments are embedded, which can be described by standard hyperelastic relations as Neo-Hookean or Mooney-Rivlin models. However, to isolate the mechanical behavior of the crosslinked actin network, the filament volume fraction is set to $\phi =1$, therefore neglecting the matrix contribution. After applying an isochoric deformation described by the deformation gradient $\bar{\textbf{F}}$, a filament initially oriented in direction $\textbf{M}$ will be deformed to a new orientation $\bar{\textbf{m}} = \bar{\textbf{F}} \cdot \textbf{M}$, as depicted in Fig. 3.

Thus, the stretch $\bar{\lambda }$ along the mean end-to-end distance is defined by

15 $$\begin{aligned} \bar{\lambda } = \sqrt{\textbf{M} \cdot \bar{\textbf{C}} \textbf{M}} = \sqrt{\bar{\textbf{m}} \cdot \bar{\textbf{m}}} ~\mathrm {.} \end{aligned}$$

The filaments are distributed over a unit sphere $\Omega$ with a relative angular density $\rho (\textbf{M})$, normalized according to

16 $$\begin{aligned} \frac{1}{4 \pi } \int _{\Omega } \rho (\textbf{M}) \, d\mathbf {\Omega } = 1 ~\textrm{,} \end{aligned}$$

with the dispersion of the crosslinked filaments $\rho (\textbf{M})$ being defined by a von Mises distribution, under rotational symmetry about the mean filament direction:

17 $$\begin{aligned} \rho (\textbf{M}) = 4 \sqrt{\frac{b}{2 \pi }} \frac{\textrm{exp}(2 b \cos ^2 \theta )}{\textrm{erfi}(\sqrt{2}b)} ~\textrm{,} \end{aligned}$$

where $\theta$ is the angle between the filament orientation and the preferential direction, while *b* is a dispersion parameter. Note that an increasing dispersion parameter *b* means the filaments are concentrated at the preferential direction, while lower *b* values lead to a more isotropic distribution. The filament force and free energy derivatives are obtained from Eqs. (4)–(9), and the free energy of the affine network is given by Holzapfel et al. (2014):

18 $$\begin{aligned} \bar{\Psi }_\textrm{AN}(\bar{\textbf{C}}, \textbf{M}) = n \int _{\Omega } \rho (\textbf{M}) \bar{w}(\bar{\lambda }) \, d\mathbf {\Omega } ~\textrm{,} \end{aligned}$$

where *n* is the number of filaments per unit volume, and $\bar{w}(\bar{\lambda })$ denotes the free energy of a single filament, which depends on the isochoric stretch $\bar{\lambda }$.

#### Constitutive relations

The application of this model to boundary value problems requires the computation of the Cauchy stress and elasticity tensors. An analogous split to Eq. (12) can be made for the Cauchy stress tensor $\boldsymbol{\sigma } = \boldsymbol{\sigma }_{\text {vol}} + \bar{\boldsymbol{\sigma }}$. The volumetric part is given by

19 $$\begin{aligned} \boldsymbol{\sigma }_{\textrm{vol}} = p^* \textbf{I}, \qquad p^{*} = \frac{\textrm{d} \Psi _{\textrm{vol}}(J)}{\textrm{d} J} \textrm{,} \end{aligned}$$

while the isochoric stress contribution relies on the fictitious Cauchy stress tensor $\tilde{\boldsymbol{\sigma }}$ and the fourth-order Eulerian projection tensor $\mathbbm {p} = \mathbb {I}^s - (\textbf{I} \otimes \textbf{I})/3$. Here, $\mathbb {I}^s$ denotes the fourth-order symmetric identity tensor, defined as $\mathbb {I}^s = \frac{1}{2}(\delta _{ik}\delta _{jl} + \delta _{il}\delta _{jk})(\textbf{e}_i \otimes \textbf{e}_j \otimes \textbf{e}_k \otimes \textbf{e}_l)$, The isochoric Cauchy stress $\boldsymbol{\bar{\sigma }}$ is then obtained as:

20 $$\begin{aligned} \boldsymbol{\bar{\sigma }} = \mathbbm {p}: \tilde{\boldsymbol{\sigma }} ~\mathrm {.} \end{aligned}$$

The fictitious Cauchy stress can also be written as the sum of the contributions from the affine network and the isotropic matrix:

21 $$\begin{aligned} \tilde{\boldsymbol{\sigma }} = \tilde{\boldsymbol{\sigma }}_{\text {AN}} + (1-\phi )\tilde{\boldsymbol{\sigma }}_{\text {IM}} ~\textrm{,} \end{aligned}$$

with each of the components being obtained by performing a push-forward operation to the fictitious 2nd Piola-Kirchhoff stress tensor $\tilde{\textbf{S}} = 2 \partial \bar{\Psi } / \partial \bar{\textbf{C}}$ in the isochoric deformation space

22 $$\begin{aligned} \tilde{\boldsymbol{\sigma }} = J^{-1} \bar{\textbf{F}} \tilde{\textbf{S}} \bar{\textbf{F}}^{\textrm{T}} ~\mathrm {.} \end{aligned}$$

Thus, the affine network stress contribution is given by

23 $$\begin{aligned} \tilde{\boldsymbol{\sigma }}_{\textrm{AN}} = n J^{-1} \int _{\Omega } \rho (\bar{\textbf{m}}) \bar{\lambda }^{-1} \bar{w}^{\prime }(\bar{\lambda }) \bar{\textbf{m}} \otimes \bar{\textbf{m}} ~\textrm{d} \Omega ~\mathrm {.} \end{aligned}$$

A similar methodology is adopted to obtain the fourth-order elasticity tensor $\mathbbm {c} = \mathbbm {c}_{\text {vol}} + \bar{\mathbbm {c}}$, with a volumetric component

24 $$\begin{aligned} \mathbbm {c}_{\textrm{vol}} = \tilde{p} \textbf{I}\otimes \textbf{I} - 2p^* \mathbb{I}^{\textrm{s}} ~\mathrm {,~~~} \tilde{p} = p^{*} + J \frac{\textrm{d}p^*}{\textrm{d}J} ~\textrm{,} \end{aligned}$$

and an isochoric component

25 $$\begin{aligned} \bar{\mathbbm {c}} = \mathbbm {p}:\tilde{\mathbbm {c}}:\mathbbm {p} + \frac{2}{3} \operatorname {tr}(\tilde{\boldsymbol{\sigma }}) \mathbbm {p} - \frac{2}{3}(\bar{\mathbf {\sigma }}\otimes \textbf{I} + \textbf{I} \otimes \bar{\mathbf {\sigma }}) ~\mathrm {.} \end{aligned}$$

Adding the contributions from the affine network and the isotropic matrix, the fictitious elasticity tensor is denoted by

26 $$\begin{aligned} \tilde{{c}} = \tilde{{c}}_{\text {AN}} + (1-\phi )\tilde{{c}}_{\text {IM}} ~\textrm{,} \end{aligned}$$

This tensor is obtained by applying the push-forward operation to the fictitious elasticity tensor in the material description $\tilde{\mathbb{C}} = 4 \partial ^2 \bar{\Psi } / \partial \bar{\textbf{C}} \partial \bar{\textbf{C}}~\textrm{}$, yielding

27 $$\begin{aligned} \tilde{\mathbbm {c}} = \textbf{F} \otimes \textbf{F} : \tilde{\mathbb {C}} : \textbf{F}^{\textrm{T}} \otimes \textbf{F}^{\textrm{T}} {~.} \end{aligned}$$

Finally, the affine network contribution to the elasticity tensor can be expressed as

28 $$\begin{aligned} \begin{aligned} \widetilde{\mathbbm {c}}_{\textrm{AN}} = n J^{-1} \int _{\Omega } \rho (\bar{\textbf{m}}) \bar{W} \bar{\textbf{m}} \otimes \bar{\textbf{m}} \otimes \bar{\textbf{m}} \otimes \bar{\textbf{m}} ~\textrm{d} \Omega ~\textrm{,} \end{aligned} \end{aligned}$$

with $$\bar{W} = \bar{\lambda }^{-2}\left[ \bar{w}^{\prime \prime }(\bar{\lambda })-\bar{\lambda }^{-1} \bar{w}^{\prime }(\bar{\lambda })\right] $$.

**Fig. 4:**
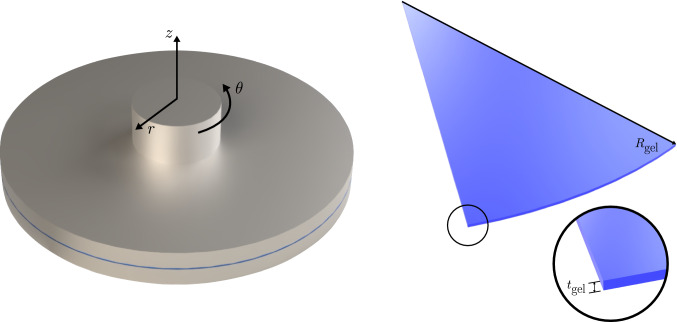
Schematic of the parallel plate rheology setup adopted for numerical implementation. A gel layer with radius $R_{\text {gel}}=20 \, \text {mm}$ and thickness $t_{\text {gel}}=140 \, \mu \text {m}$ is placed between and tied to the rigid plates. Rotation of the top plate imposes a maximum shear strain of $\mathrm \gamma =0.25$ at the outer edge of the gel

Numerical integration of Eqs. (23) and (28) is carried out by discretizing the unit sphere into spherical triangles following Li et al. (2018), with each triangle centroid taken as a filament direction. A total of 720 centroid directions were employed for the quadrature. To justify the choice of this discretization, a convergence study was performed on a unit cube under simple shear in Abaqus, by running 10 000 simulations with varying material parameters. The analysis showed that 720 directions achieved results comparable to the finest discretization tested (2880 directions), with relative errors in the mean and standard deviation (SD) of the shear and normal stresses remaining below 0.1%. This ensures the numerical integration errors are negligible compared to the stochastic variability of the material.

Due to the semiflexible nature of F-actin filaments, they are regarded as being unable to support compressive loads. Therefore, the filaments are assumed to be active in extension while becoming inactive under compression (Holzapfel et al. 2000; Alastrué et al. 2009). As a result, they only contribute to the network stress and elasticity tensors when $\bar{\lambda } \ge 1$.

To ensure consistency of the finite element implementation, it was verified against a semi-analytical solution for simple shear deformation, as it represents the predominant loading condition in soft tissue rheology. The detailed verification results and the comparison between the numerical and semi-analytical solutions are provided in Appendix A.

### Numerical implementation

As a first numerical example, we implemented the material model in a cubic element of unitary length ($1 \, \mu \text {m}$). The unit cube is subjected to fundamental deformation modes: simple shear, uniaxial and biaxial extension. For the first mode, a maximum shear strain $\gamma = 0.25$ is applied, while for the remaining, a maximum stretch of $\lambda = 1.15$ is imposed, allowing the material to reach the nonlinear regime.

A representative model of the parallel plate bulk rheology setup described in Shin et al. (2004) was created, focusing only on the nonlinear elastic response of the gel layer. Figure 4 shows the scheme of the setup, with a thin gel layer of radius $R_{\text {gel}}= 20 \, \text {mm}$ and thickness $t_{\text {gel}} = 140 \, \mu \text {m}$ placed between the rigid plates. Only a $30^\circ$ section of the gel layer is modeled, being tied to both top and bottom rigid plates. A rotation is imposed to the top plate such that a maximum shear strain of $\gamma = 0.25$ is reached at the outer edge. A mesh with 3226 hexahedral elements (Abaqus’ C3D8) is used to discretize the gel layer. A single element layer is employed through the thickness. The remaining mesh is designed to provide an in-plane characteristic length scale comparable to the gap height, with 100 elements in the radial direction and 52 elements along the outer circumferential edge, therefore increasing resolution near the outer periphery where peak stresses occur.

Regarding the element formulation, the volumetric constraint is enforced by a compressible formulation with the penalty term in Eq. (13), where a finite bulk modulus *k* is used to penalize volume changes. This approach effectively enforces near-incompressibility without imposing strict kinematic constraints ($J=1$) that can lead to numerical ill-conditioning. This is particularly relevant because in strict incompressibility regimes, standard displacement-based elements can lead to volumetric locking. To prevent this, mixed formulations - where pressure is treated as an independent degree of freedom - are typically required (hybrid elements in Abaqus). However, the standard displacement formulation remains valid in this work, as the penalty term mitigates locking phenomena, the unit cube benchmark is consistent with the semi-analytical solution (Appendix A), and the rheology simulations produced identical results when compared against hybrid element formulations.

An experimental design space of 10 000 samples was created with Latin Hypercube Sampling (LHS), varying six material properties of the compound filament - $r_{0,c}$, $\eta$, $\mu _0$, $L_p$, *L* and $r_{0,f}$ - according to the same distributions used for the construction of the surrogate model in Sect. 2.4. As for the network parameters, we set a fixed filament density of $n = 7.66 \, \mu \text {m}^{-3}$. For the contour length and initial end-to-end distance adopted in this work, this choice satisfies the filament density relations reported by Palmer and Boyce (2008), and Unterberger et al. (2013a). Moreover, a low dispersion parameter $b = 0.001$ was selected to approximate an isotropic distribution ($\rho \approx 1$). While a mean orientation $\textbf{M} = \textbf{E}_1$ was defined, its influence is negligible in this limit. For each of the four numerical examples, the full experimental design of 10 000 LHS samples was simulated. The resulting responses were taken as reference for the assessment of the surrogate model’s accuracy.

### PCE surrogate modeling

A surrogate model $\tilde{\mathcal {M}}$ is an approximation of the original model $\mathcal {M}$ that is computationally cheaper to evaluate. It is built from an experimental design space $\mathcal {X} = \{\textbf{x}^{(i)}, i=1, \dots , N\}$ - a limited set of runs on the original model $\mathcal {M}$ - assuming some regularity on the model $\mathcal {M}$ and some general functional shape. The experimental design $\mathcal {X}$ must be selected so that it adequately covers the admissible region of the input-parameter space, using methods as MCS, LHS or low-discrepancy sequences. One of the most common approaches for building a surrogate model is the PCE, which allows for the representation of a quantity of interest *Y* with a surrogate $\tilde{\mathcal {M}}(\textbf{X})$, where $\textbf{X}$ is the vector of *M* random input parameters, $\textbf{X} \in \mathbb {R}^M$. Therefore, the quantity of interest can be expressed as

29 $$\begin{aligned} Y = \tilde{\mathcal {M}}(\textbf{X}) = \sum _{\alpha \in \mathbb {N}^M} q_{\alpha } \Phi _{\alpha }(\textbf{X}), \end{aligned}$$

where $q_{\alpha }$ are scalar coefficients to be computed and $\Phi _{\alpha }(\textbf{X})$ are scalar-valued basis functions (orthonormal polynomials). While MCS explores the output space and distribution point by point, the PCE explores it through a polynomial function. It studies the contribution of *N* basis surfaces and sums them. The methodology for constructing a PCE surrogate can be summarized in a few steps. First, the polynomial basis functions $\Phi _{\alpha }(\textbf{X})$ must be constructed, for a distribution function $f_{X_i}$. Then, the coefficients $q_{\alpha }$ are computed and the accuracy of the expansion is evaluated. Finally, uncertainty quantification and sensitivity analysis post-processing can be efficiently carried out based on the polynomial obtained.

In this work, the PCE surrogate will replace the original mechanical model of crosslinked F-actin filaments in the network model. Thus, the quantities of interest or model outputs are the filament force *f*, and the first and second derivatives of the filament strain energy, $w^\prime$ and $w^{\prime \prime }$, respectively, which are required for network-scale computations. Therefore, the vector of output quantities is $\textbf{Y} = \{f, w^\prime , w^{\prime \prime }\}$.

#### Basis functions

In a problem with several random inputs, a joint probability density function (PDF) can be defined:

30 $$\begin{aligned} f_X(\textbf{x}) = \prod _{i=1}^M f_{X_i}(x_i) ~\mathrm {.} \end{aligned}$$

For each marginal distribution function $f_{X_i}(x_i)$, a family of orthogonal polynomials $\{P_k^{(i)}, \, k \in \mathbb {N}\}$ is built:

31 $$\begin{aligned} \begin{aligned}&\left\langle P_j^{(i)}(x_i), P_k^{(i)}(x_i) \right\rangle = \\&=\int P_j^{(i)}(x_i) P_k^{(i)}(x_i) f_{X_i}(x_i) \, dx_i = \gamma _j^{(i)} \delta _{jk} ~\mathrm {.} \end{aligned} \end{aligned}$$

Each family of orthogonal polynomials (Legendre, Hermite, Laguerre) is associated with a specific probability distribution function (uniform, Gaussian, gamma, respectively) and the PCE basis recurrence formula depends on the family of polynomials chosen. There are many ways to compute the orthogonal polynomials for each random variable, based on its distribution function. In this work, the three-term recurrence relation was used, as it is numerically stable and because only independent distributions are considered.

The number of random variables *M* allows defining the multi-indices $\alpha = \{\alpha _1, \dots , \alpha _M\}$ of degree $|\mathbf {\alpha }| = \sum _{i=1}^{M} \alpha _i$, leading to the corresponding multivariate polynomial (see, e.g.,(Blatman and Sudret 2011; Feinberg and Langtangen 2015)):

32 $$\begin{aligned} \Phi _{\alpha }(\textbf{X}) = \prod _{i=1}^M \Phi _{\alpha _i}^{(i)}(X_i) ~\mathrm {.} \end{aligned}$$

When multiplied by coefficients $q_{\alpha }$, the PCE representation of a quantity of interest (Eq. (29)) is achieved. Since an infinite series expansion cannot be handled in practical computations, a truncation of the polynomial is necessary. Considering multivariate polynomials of total degree $|\mathbf {\alpha }| = \sum _{i=1}^{M} \alpha _i$, less than or equal to a given *p*:

33 $$\begin{aligned} \mathcal {A}^{M,p}= \{ \alpha \in \mathbb {N}^M: |\alpha | \le p \} \end{aligned}$$

34 $$\begin{aligned} P = \left( {\begin{array}{c}M + p\\ p\end{array}}\right) = \frac{(M + p)!}{M! p!} ~\textrm{,} \end{aligned}$$

where *P* is the number of terms in the PCE, which depends on the number of random input variables and the polynomial order *p*.

**Fig. 5:**
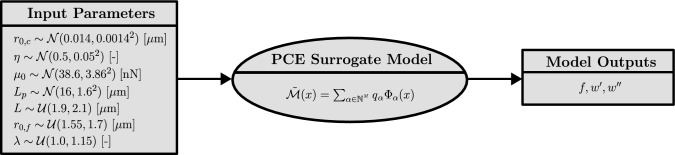
Summary of the PCE surrogate model, detailing input distributions and model outputs

The surrogate model for crosslinked F-actin depends on 7 input parameters. Following Eq. (34), the polynomial basis size grows combinatorially with the degree, increasing from 330 terms at $p=4$ to 792 terms at $p=5$, and reaching 1716 terms at $p=6$. Hence, we adopt a sparse PCE approach that retains only the most significant basis functions and set the maximum polynomial degree to $p=5$. This truncation was selected to limit the expansion of the candidate basis while ensuring reliable local accuracy, thus reducing the surrogate’s computational cost without sacrificing relevant higher-order contributions. The scheme in Fig. 5 summarizes the distributions of the input parameters and the quantities of interest that must be obtained for the surrogate model to be implemented in the UMAT framework.

The probability distributions for the input parameters were selected to reflect experimental observations and physical constraints, with mean values adopted from Holzapfel et al. (2014). Material parameters were generally modeled as Gaussian distributions, $X \sim \mathcal {N}(\mu ,\sigma ^2)$, with a standard deviation set to 10% of the mean value, i.e., $\sigma = 0.1 \mu$. This approach normalizes the sensitivity analysis while capturing the experimental uncertainty and biological variability typical of the literature. For the stretch modulus $\mu _0$, the mean value was set to 38.6 nN, consistent with measurements of $43.7 \pm 4.6$ nN reported by Kojima et al. (1994) and $35 \pm 4$ nN by Matsushita et al. (2010). The assumed 10% standard deviation creates a distribution that effectively captures the mean values reported in these studies, placing them within a statistically plausible range of the adopted mean. Similarly, the persistence length $L_p$ was centered at 16 $\mu$m, aligned with reported values of $17.7 \pm 1.1~\mu$m (Gittes et al. 1993), 16 $\mu$m (Janmey et al. 1994), and 15 $\mu$m (Isambert and Maggs 1995).

The initial crosslinker length $r_{0,c}$ depends on the species involved in the network. Reported dimensions range from $\approx 8$ nm for fascin (Jansen et al. 2011) and $\approx 12$ nm for fimbrin (Volkmann et al. 2001) to larger spans such as $33.9 \pm 2.5$ nm for $\alpha$-actinin (Hampton et al. 2007) and $\approx 160$ nm for filamin (Nakamura et al. 2011). We adopted a mean value in the lower range, as nanometer-scale variations do not significantly influence the filament response. The relative stiffness $\eta$ is a phenomenological parameter lacking a direct experimental fit. Given that over 60 distinct actin-binding proteins have been identified (Shin et al. 2004), including both compliant and rigid species, it is plausible to model their aggregate random effect as converging to a normal distribution.

In contrast, exceptions were made for the filament length *L* and end-to-end distance $r_{0,f}$, which were treated as uniform distributions ($\mathcal {U}$). This approach enables the explicit enforcement of the constraint $r_{0,f} < L$, ensuring physically valid networks. The adopted mean value $L = 2 {~}\mu$m is consistent with both the length of gelsolin-capped filaments reported by Janmey et al. (1994) and the value derived from the network relations in Holzapfel et al. (2014). Finally, the mean $r_{0,f}$ of approximately 1.63 $\mu$m maintains a suitable ratio with *L*, preventing a high number of samples where filaments would excessively exceed their contour length under the considered stretch range. While a physical correlation likely exists between these parameters (bounded by $r_{0,f} < L$), sufficient experimental data to quantify their joint probability density function is currently lacking. Therefore, the assumption of independence was maintained to avoid introducing unfounded bias into the surrogate model, relying instead on the defined range limits to avoid unphysical samples. To ensure an even coverage of the deformation space, the stretch $\lambda$ was also assumed to be uniformly distributed within the range [1.0, 1.15]. The lower bound is set to 1.0, as the contribution of compressed filaments is excluded. This interval allows exploring the highly nonlinear regime of the filament response, while avoiding extreme variations in quantities of interest that the PCE surrogate would not be able to capture.

#### Computing PCE coefficients

Methods for computing PCE coefficients can be divided into two main categories: intrusive and non-intrusive approaches. Projection of the equations in the Galerkin sense is an example of the first kind, requiring modifications to the underlying deterministic model. In contrast, non-intrusive approaches, such as the point-collocation method (Tatang et al. 1997; Campos et al. 2019) adopted in the present work, treat the computational model as a black box. Such a framework relies on a design of experiments to sample the input space, requiring only the model’s output at discrete points to construct the surrogate. This experimental design is used to compute the coefficients of the PCE basis functions using methods such as least squares minimization. To do so, the exact (infinite) series expansion is considered as the sum of a truncated series and a residual term $\varepsilon _P$:

35 $$\begin{aligned} \begin{aligned} Y = \mathcal {M}(\textbf{X})&= \sum _{\alpha \in \mathcal {A}} q_{\alpha } \Phi _{\alpha }(\textbf{X}) + \varepsilon _P = \\&= \mathbf {Q^T \Phi (X)} + \varepsilon _P(\textbf{X}) \end{aligned} \end{aligned}$$

where:

36 $$\begin{aligned} \begin{aligned} \textbf{Q}&= \{ q_\alpha , \mathbf {\alpha } \in \mathbf {\mathcal {A}} \} = \{q_0, \dots , q_{P-1}\} \\ \mathbf {\Phi (X)}&= \{ \Phi _0(\textbf{X}), \dots , \Phi _{P-1}(\textbf{X}) \} \end{aligned} \end{aligned}$$

and the residual associated with the finite polynomial with *P* terms is computed as

37 $$\begin{aligned} \varepsilon _P(\textbf{X}) = \mathcal {M}(\textbf{X}) - \sum _{j=0}^{P-1} q_{j} \Phi _{j}(\textbf{X}) ~\mathrm {.} \end{aligned}$$

The unknown coefficients $\textbf{Q}$ are computed by minimizing the mean square residual error:

38 $$\begin{aligned} \begin{aligned} \textbf{Q}&= \mathop {\textrm{arg min}}\limits _{q \in \mathbb {R}^{P}} \mathbb {E} \left[ \varepsilon _P^2(\textbf{X}) \right] \\&= \mathop {\textrm{arg min}}\limits _{q \in \mathbb {R}^{P}} \mathbb {E} \left[ \left( \mathcal {M}(\textbf{X}) - \textbf{Q}^T \mathbf {\Phi (X)} \right) ^2 \right] \end{aligned} \end{aligned}$$

with $\mathbb {E}[\cdot ]$ denoting the expectation operator with respect to the probability distribution of the random input parameters. By sampling the input space with *N* realizations and computing the corresponding model evaluations, the minimization problem in Eq. (38) can be approximated by the discrete least-squares problem:

39 $$\begin{aligned} \hat{\textbf{Q}} = \mathop {\textrm{arg min}}\limits _{q \in \mathbb {R}^{P}} \frac{1}{N} \sum _{i=1}^N \left( \mathcal {M}(\textbf{x}^{(i)}) - \textbf{Q}^T \mathbf {\Phi }(\textbf{x}^{(i)}) \right) ^2 ~\mathrm {.} \end{aligned}$$

The estimate for the coefficients is obtained by solving Eq. (39), leading to the following linear system in matrix form:

40 $$\begin{aligned} \hat{\textbf{Q}} = (\textbf{A}^T \textbf{A})^{-1} \textbf{A}^T \textbf{M} \end{aligned}$$

where $\textbf{A}$ is the experimental matrix of size $N \times P$, and $\textbf{M}$ is a column vector of size *N* containing the model evaluations. The ordinary least-squares (OLS) approach is straightforward and computationally efficient for low-order expansions, but accurately capturing complex model responses often requires higher polynomial degrees. As the polynomial order *p* increases, the number of model evaluations *N* needed to ensure an accurate estimation of the coefficients also increases significantly. Moreover, OLS yields a dense coefficient vector that retains meaningless high-order terms, which raises computational cost, while promoting overfitting and higher variance.

To overcome these limitations, a sparse PCE was developed. There are several techniques to obtain a sparse PCE (Lüthen et al. 2021), with the most common being Orthogonal Matching Pursuit (OMP) (Tropp and Gilbert 2007) and Least Angle Regression (LARS) (Efron et al. 2004). OMP is a forward greedy method that starts with an empty model and iteratively selects the basis function with the highest correlation with the current residual, adding it to the set of active basis functions. The coefficients are updated at each iteration using OLS on the active set. LARS is also a forward greedy technique that builds the model incrementally, but unlike OMP, it updates the coefficients such that all active regressors have equal correlation with the residual. The coefficients are moved in an equiangular direction until a nonactive basis function reaches the same correlation with the residual as the active ones, at which point it is added to the active set. By accounting for correlations among basis functions, it enables more effective basis selection than strictly greedy methods. Therefore, we employed LARS regression to construct the PCE surrogates, as it generally provides better accuracy with fewer basis functions.

An experimental design space with 6000 samples (80% for training and 20% for testing) was generated using LHS, following the distributions in Fig. 5. For each sample, the quantities of interest $\textbf{Y} = \{f, w^\prime , w^{\prime \prime }\}$ were computed using the mechanical model of crosslinked F-actin filaments.

#### PCE post-processing

From the orthonormality of the polynomial basis, we have

41 $$\begin{aligned} \mathbb {E} \left[ \Phi _\alpha (\textbf{X}) \right] = 0 ~{~~} \end{aligned}$$

42 $$\begin{aligned} \mathbb {E} \left[ \Phi _\alpha (\textbf{X}) \Phi _\beta (\textbf{X}) \right] = 0 ~{,~~~} \alpha \ne \beta \end{aligned}$$

so that the mean value is the zero-th order coefficient: $\hat{\mu }_Y=q_0$. The variance of the quantity of interest *Y* can be computed as:

43 $$\begin{aligned} \hat{\sigma }_Y^2 = \mathbb {E} \left[ \left( Y^{PC} - \hat{\mu }_Y \right) ^2 \right] = \mathbb {E} \left[ \left( \sum _{\alpha \in \mathcal {A} \setminus \textbf{0}} q_{\alpha } \Phi _\alpha (\textbf{X}) \right) ^2 \right] \nonumber \\ \end{aligned}$$

which simplifies to the sum of the squares of all coefficients, except the bias term. Thus, the standard deviation is expressed as $\hat{\sigma }_Y = \sqrt{\sum _{\alpha \in \mathcal {A} \setminus \textbf{0}} q_{\alpha }^2}$.

Sobol’ indices are a measure of the influence of each input parameter on the quantities of interest. 1^st^-order Sobol’ indices $S_i$ measure the additive effect of each input parameter separately, while total Sobol’ indices $S^T_i$ quantify the total effect of each input parameter, including interactions with other variables. Their computation is straightforward from the PCE coefficients, so that the 1^st^-order Sobol’ indices result in

44 $$\begin{aligned} S_i = \frac{1}{\hat{\sigma }_Y^2} \sum _{\alpha \in \mathcal {A}_i} q_{\alpha }^2 ~{,} \end{aligned}$$

while the total Sobol’ indices, considering the set $\mathcal {A}_i^T = \{ \alpha \in \mathcal {A}: \alpha \supset i \}$, are given by

45 $$\begin{aligned} S_i^T = \frac{1}{\hat{\sigma }_Y^2} \sum _{\alpha \in \mathcal {A}_i^T} q_{\alpha }^2 ~{. } \end{aligned}$$

While Eqs. (41)–(45) establish the global statistical moments and sensitivity indices over the complete joint probability space, the results presented throughout Sect. 3 are interpreted as conditional statistics. Since the stretch $\lambda$ is included as an input parameter to allow a single surrogate to represent the filament response over the deformation range, such quantities are obtained by conditioning the PCE on specific realizations of $\lambda$, and evaluating the surrogate with respect to the remaining stochastic input parameters. In the FE implementation, the UMAT subroutine evaluates the moment-based surrogate outputs - filament force and the derivatives of the free energy - to assemble the network stress and the corresponding elasticity tensor.

## Results and discussion

### Filament surrogate validation

Before integrating the filament surrogate within the FEM framework, its predictions are validated against the test dataset from the mechanical model. In addition, we perform uncertainty quantification of the quantities of interest and a global sensitivity analysis of the material parameters.

To select the appropriate polynomial order, the model’s performance was assessed using two error metrics: the mean absolute percentage error (MAPE) and the normalized root-mean-square error, $\text {NRMSE} = \frac{\text {RMSE}}{(y_{\text {max}}-y_{\text {min}})}$. The NRMSE proved to be a relatively insensitive metric for model selection. Even when identifying the candidate models that yielded the lowest values for this metric, they remained in a narrow range of 0.01 to 0.02, across $p=3$ to 5. This occurs because the NRMSE is dominated by the high-magnitude regions, resulting in low global error scores despite significant relative discrepancies in the lower range of the quantities of interest. As such, the MAPE served as the distinguishing criterion.

Table 1 summarizes the accuracy for the models selected based on the MAPE. The results highlight the need for 5^th^-order expansion, since the gain in local predictions is substantial. For instance, increasing the degree from $p=3$ to $p=5$ reduces the MAPE for the filament force *f* from 56.2% to 8.9%, confirming that higher-order terms are critical for an accurate characterization of the response. Through this sparse selection, the final surrogates ($p=5$) retain only 87 coefficients for the three quantities of interest. This represents a significant reduction from the 792 terms required for a full 5^th^-order expansion, ensuring a compact representation that does not overfit the training data.

**Table 1:** Filament surrogate evaluation metrics (MAPE and NRMSE) on the test set, for the quantities of interest *f*, $w^{\prime }$, and $w^{\prime \prime }$. Results are shown for polynomial degrees $p=3$ to 5, corresponding to the configurations that minimized the MAPE

	*f*		$w^{\prime }$		$w^{\prime \prime }$	
*p*	MAPE	NRMSE	MAPE	NRMSE	MAPE	NRMSE
3	56.2%	0.025	49.5%	0.061	8.1%	0.037
4	25.0%	0.018	26.8%	0.018	6.3%	0.028
5	8.9%	0.010	9.5%	0.011	3.4%	0.015

**Fig. 6:**
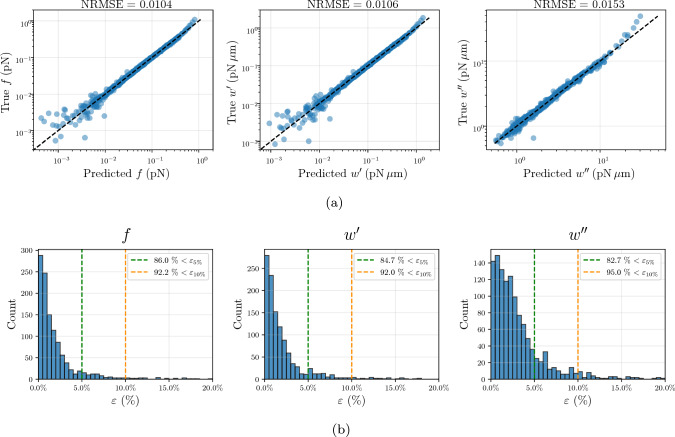
Filament surrogate predictions on a 1200-sample test set. (**a**) True vs predicted values for the three quantities of interest: *f*, $w^\prime$, and $w^{\prime \prime }$. (**b**) Histograms of absolute percentage error $\varepsilon$, highlighting the percentage of predictions with errors below 5% ($\varepsilon _{5\%}$) and 10% ($\varepsilon _{10\%}$

The validation of these selected surrogates on the 1200-sample test set is detailed in Fig. 6. Consistent with the selection metrics, the global NRMSE remains low and similar across all three quantities of interest (Fig. 6a). However, as indicated by the discrepancy between global and local metrics, the largest relative errors for the filament force *f* and the first derivative $w^\prime$ of the free energy typically occur for values below $10^{-2}$. This is a consequence of the PCE construction, which minimizes the mean-squared error, a metric that is dominated by high-magnitude values. While a logarithmic transformation of the output could improve relative accuracy in this region, a linear-scale surrogate was maintained to preserve the ability to analytically derive statistical moments and Sobol’ sensitivity indices directly from the expansion coefficients. Nevertheless, in the context of the full network, these near-null filament responses would contribute almost negligibly to the overall mechanical behavior, particularly as deformation increases, so inaccuracies in this region have limited practical impact on the predicted macroscopic response. The second derivative $w^{\prime \prime }$ is less accurately predicted only for a few high-value outliers, above $10^1$, due to the high variation of this quantity over the considered stretch range, making extreme values harder to capture with a single PCE surrogate. In this region, the model tends to underpredict the response, attributed to the regularization inherent in the sparse basis selection, which restricts complexity to minimize the generalization error. By prioritizing a robust global approximation, the PCE avoids overfitting to these rare responses. As a result, such discrepancies comprise only a small fraction of the test set: the histograms of Fig. 6b show that for the three model outputs, over 80% of the predictions have an absolute percentage error $\varepsilon < 5\%$, and more than 90% have $\varepsilon < 10\%$.

**Fig. 7:**
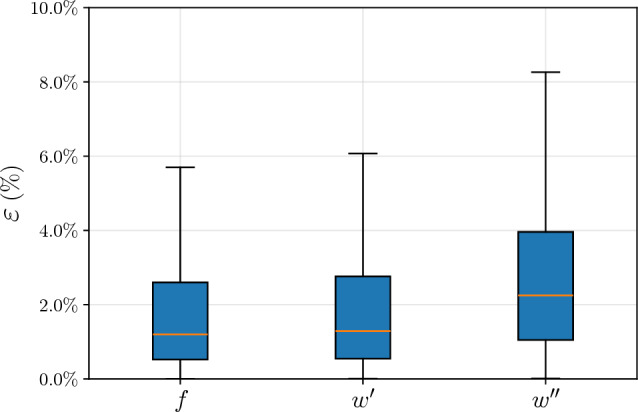
Boxplots of absolute percentage error $\varepsilon$ for the three quantities of interest

To further complement the validation, the box plots in Fig. 7 illustrate that the median $\varepsilon$ remains below or very close to 2% across all three quantities of interest, while the third quartile is below 4%.

**Fig. 8:**
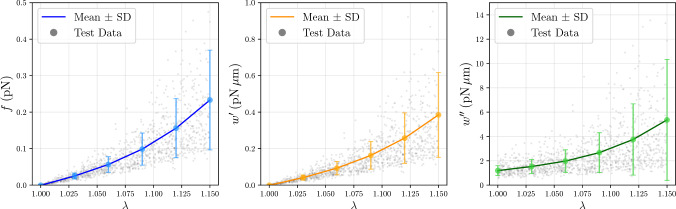
Mean and standard deviation of quantities of interest across the full stretch domain. Gray dots represent the test set samples

Uncertainty quantification of the PCEs was performed for all quantities of interest, *f*, $w^\prime$ and $w^{\prime \prime }$. By leveraging the orthogonality of the polynomial basis, the statistical moments were derived directly from the expansion coefficients. Figure 8 shows the mean curve with standard deviation bands for each quantity, along the entire stretch domain. The superimposed test data points predominantly fall within the statistical bounds, indicating that the surrogate effectively captures the variability in the model outputs. Table 2 summarizes the mean, SD and coefficient of variation (CoV) for two stretch values, $\lambda = 1.05$ and $\lambda = 1.1$. As expected, the standard deviation increases with stretch, which is compatible with the nonlinear nature of the mechanical model. In addition, the coefficient of variation is also higher for higher stretches, indicating that the filament response becomes less predictable with increasing deformation.

Due to their linear relation, it is noticeable that there is a close agreement between the standard deviation and coefficient of variation of *f* and $w^\prime$, for both stretch values. In contrast, despite the coefficient of variation of $w^{\prime \prime }$ being similar to that of *f* and $w^\prime$ at $\lambda = 1.05$, it is significantly higher for greater stretch values, indicating that the second derivative of the free energy is more sensitive to uncertainty in the input parameters.

**Table 2:** Mean, standard deviation, and coefficient of variation of the quantities of interest at $\lambda =1.05$ and $\lambda =1.1$

	$\lambda = 1.05$			$\lambda = 1.1$		
	*f* [pN]	$w'$ [pN.$\mu$ m]	$w''$ [pN.$\mu$ m]	*f* [pN]	$w'$ [pN.$\mu$ m]	$w''$ [pN.$\mu$ m]
Mean	0.0450	0.0740	1.8044	0.1158	0.1911	2.9802
SD	0.0161	0.0278	0.7875	0.0545	0.0935	2.0016
CoV	35.86%	37.53%	43.64%	47.08%	48.92%	67.16%

**Fig. 9:**
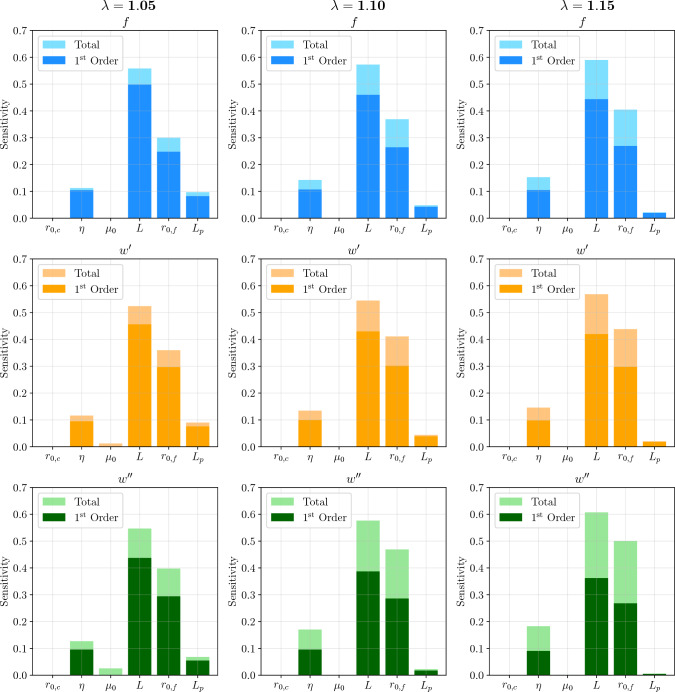
First-order and total Sobol’ indices for the quantities of interest in three stretch values

Finally, a sensitivity analysis was conducted to identify the most influential material parameters on the PCE outputs, and the ones that could be modeled as deterministic constants without significantly affecting the surrogate accuracy. First-order and total Sobol’ indices are computed for the quantities of interest at three stretch values, $\lambda = 1.05$, $\lambda = 1.1$, and $\lambda = 1.15$, as depicted in Fig. 9. The applied stretch $\lambda$, despite being included as an input in the PCE surrogates, is not accounted for in the sensitivity analysis, as it is not a material parameter but rather a deformation descriptor.

For all model outputs and for the distributions and intervals considered in this work, the Sobol’ indices indicate that the most influential material properties are the filament contour length *L* and the filament initial end-to-end distance $r_{0,f}$. Mechanical models for biopolymer filaments clearly indicate that the filament response becomes strongly nonlinear as the filament end-to-end distance approaches the contour length, which explains the high sensitivity to these quantities. This is captured in the Sobol-based sensitivity analysis, which shows that the interaction between these two parameters is very significant for the filament response, and increases with stretch, as shown by the growing total Sobol’ indices, while the first-order indices remain relatively constant for $r_{0,f}$ and decrease slightly for *L*. In contrast, the crosslinker initial end-to-end distance $r_{0,c}$ and filament stretch modulus $\mu _0$ have negligible influence on the outputs, which could allow them to be treated as constant values in PCE surrogate representations, instead of being modeled by a probabilistic distribution. This observation is consistent with Holzapfel et al. (2014), where it was noted that varying $r_{0,c}$ within the nanometer range produces no significant impact on the results. Despite its role in the filament mechanical equations, the negligible influence of the stretch modulus $\mu _0$ - representing the enthalpic contribution - is consistent with the reported behavior of semiflexible polymers. The compliance of a filament is governed by a competition between enthalpic stretching and the entropic straightening of thermal fluctuations. For F-actin, the response is dominated by these thermal fluctuations as long as the contour length *L* satisfies the condition $L^3 \gtrsim a^2 L_p$ (Broedersz and MacKintosh 2014), where $a \approx 3.5 {~nm}$ is the radius of actin filaments. Given the mean contour length ($L=2~\mu \text {m}$) and persistence length ($L_p=16~\mu \text {m}$) adopted in this study, we verify that $L^3 \gg a^2 L_p$, placing the filaments strictly within the entropic regime. This explains the negligible influence of the stretch modulus $\mu _0$ on the filament response within the considered stretch range, where the end-to-end distance has not yet reached the contour length. Additionally, even for $r = L$, Unterberger et al. (2013a) reported that the influence of the stretch modulus remains secondary to that of the characteristic geometric lengths. The crosslinker relative stiffness $\eta$ and persistence length $L_p$ also have some influence on those 3 outputs, but with contrary effects. While $\eta$ increases its influence with stretch, mainly when accounting for its interactions with other parameters, $L_p$ has a decreasing effect, which is likely due to the fact that the persistence length is a measure of the filament bending stiffness, thus having a less pronounced effect when the filament is already stretched. Finally, the dominance of *L* and $r_{0,f}$ in the sensitivity analysis underscores the potential impact of their statistical dependence. While the current framework captures the mechanical interaction between these parameters, it does not employ a copula to explicitly enforce a probabilistic correlation. Modeling this dependence through standard isoprobabilistic transformations (e.g., Rosenblatt or Nataf) often introduces strong nonlinearities that degrade the convergence of the polynomial approximation (Jakeman et al. 2019). Alternatively, constructing a basis consistent with the joint density via standard Gram-Schmidt orthogonalization is known to be numerically ill-conditioned. While recent data-driven methodologies have been proposed to stabilize these expansions and address such computational challenges (Liu and Choe 2023; Zeng and Ghanem 2025), their successful implementation relies on the availability of experimental data to define the correlation structure.

### PCE-FEM implementation

The PCE-FEM implementation involves substituting the filament equations with a surrogate model in the network-level framework (leading to the Network Surrogate), which allows for a faster model description, thereby replacing lengthy MCS. The Network Surrogate provides the first two statistical moments of the three quantities of interest at the filament scale, conditioned on the stretch of each filament orientation in an integration point. Each moment requires a separate surrogate evaluation, therefore obtaining the higher-scale statistical information requires two model runs. Results are validated against 10 000 MCS of the original mechanical implementation on a unit cube element, performed for each of three loading conditions: simple shear, uniaxial extension, and biaxial extension.

**Fig. 10:**
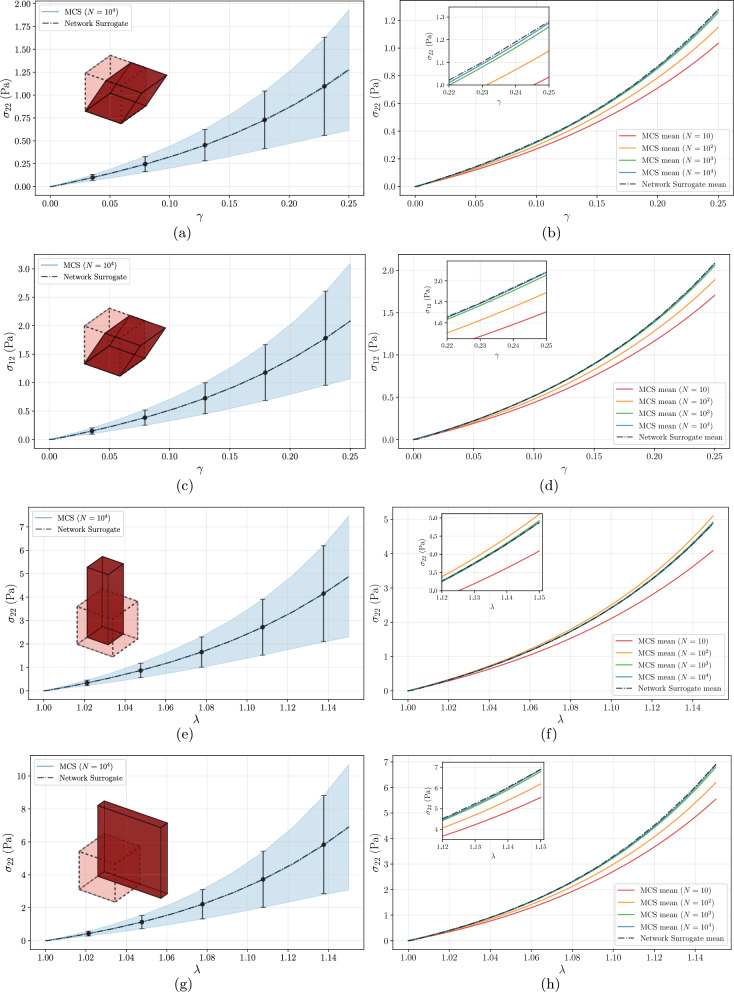
Comparison between MCS and Network Surrogate results for different loading cases in the unit cube FEM example: simple shear (a-d), uniaxial extension (e-f), and biaxial extension (g-h). Left-column plots (a,c,e,g) show mean value and standard deviation bands from MCS with $N=10^4$ samples and the Network Surrogate. Right-column plots (b,d,f,h) compare the MCS mean for increasing sample sizes with the Network Surrogate mean

For the simple shear case, with a maximum shear strain of $\gamma =0.25$, the Network Surrogate presents excellent agreement with the MCS results, as shown in Fig. 10(a,c). The Network Surrogate expected curve is almost coincident with the MCS mean curve, and the standard deviation is accurately captured for all stretch values and for both stress components.

In Fig. 10(b,d), the mean curve from the Network Surrogate is plotted alongside MCS mean curves for increasing sample sizes ($N=10$, $N=10^2$, $N=10^3$ and $N=10^4$). It is noticeable that the MCS mean curves converge toward the Network Surrogate simulation as the sample size increases. Table 3 shows that the Network Surrogate, regarding the expected stress values for the maximum shear considered ($\gamma =0.25$), produces better results than MCS with $N=10^3$ samples, with relative errors $\varepsilon$ of 0.15% for the shear stress $\sigma _{12}$ and 0.55% for the normal stress $\sigma _{22}$, when compared to the reference solution obtained with $10^4$ MCS samples. Consistent with the entropic filament nature, the network-level response exhibits characteristic strain-stiffening behavior. The shear modulus ($\partial \sigma _{12} / \partial \gamma$) increases by a factor of 3 to 4 at the maximum applied shear, for the mean response and upper bound of the standard deviation. While extreme stiffening is predicted near network rupture, the observed magnitude is consistent with theoretical (Broedersz et al. 2008) and experimental (Gardel et al. 2004) benchmarks for this deformation range, confirming that the model accurately captures the onset of the entropic nonlinear regime.

**Table 3:** Mean values and absolute percentage errors ($\varepsilon$) of the shear stress $\sigma _{12}$ and normal stress $\sigma _{22}$ at maximum deformation: $\gamma =0.25$ for simple shear, $\lambda =1.15$ for uniaxial and biaxial loading cases. Results are shown for different MCS sample sizes compared with the Network Surrogate. The MCS solution with $N=10^4$ serves as the reference

	Simple Shear $\gamma =0.25$		Uniaxial $\lambda =1.15$	Biaxial $\lambda =1.15$
	$\sigma _{12}$	$\sigma _{22}$	$\sigma _{22}$	$\sigma _{22}$
	Mean (Pa) [$\varepsilon$]	Mean (Pa) [$\varepsilon$]	Mean (Pa) [$\varepsilon$]	Mean (Pa) [$\varepsilon$]
$N = 10^4$	2.081 [ - ]	1.274 [ - ]	4.879 [ - ]	6.891 [ - ]
$N = 10^3$	2.056 [1.35%]	1.257 [1.84%]	4.924 [0.91%]	6.794 [1.42%]
$N = 10^2$	1.888 [9.42%]	1.150 [10.19%]	5.101 [4.54%]	6.192 [10.15%]
$N = 10$	1.705 [18.19%]	1.035 [19.21%]	4.090 [16.17%]	5.541 [19.61%]
Network Surrogate	2.084 [0.15%]	1.281 [0.55%]	4.871 [0.17%]	6.903 [0.16%]

Uniaxial stretching simulations accounted for a maximum stretch of $\lambda _{22} = 1.15$. The results for the normal stress $\sigma _{22}$ along the deformation history are shown in Fig. 10e, where it is once again evident that the Network Surrogate accurately captures the MCS mean curve and variability. The expected value curves for the network surrogate, MCS with $N=10^3$ and $N=10^4$ almost superpose, as displayed in Fig. 10f. This is confirmed by the data presented in Table 3, for the maximum stretch value of $\lambda _{22}=1.15$, where both methods yield a relative error below 1%. However, the Network Surrogate is still able to outperform the results from 1000 MCS samples.

Similar to the uniaxial case, biaxial extension simulations were performed for a maximum stretch of $\lambda _{11} = \lambda _{22} = 1.15$. The plots depicted in Fig. 10g, for the normal stress $\sigma _{22}$ throughout the stretching history, confirm that the Network Surrogate captures the MCS expected values with great accuracy. The predictions for the standard deviation are also very close to the MCS results.

The comparison of the Network Surrogate mean curve with MCS mean results with increasing sample sizes (Fig. 10h) displays a similar trend to the previous cases, in the sense that the MCS results converge toward the Network Surrogate. The inset plot highlights the close agreement between the Network Surrogate and MCS with $N=10^3$ and $N=10^4$ samples at the higher range of deformation. For the maximum stretch, the Network Surrogate provides once again the closest expected value to the reference solution ($\varepsilon = 0.16\%$), as shown in Table 3.

Across all deformation modes, the Network Surrogate maintains relative errors below or near 1% for the majority of the deformation range. However, accuracy decreases in the very low deformation regime ($\gamma <0.025$ and $\lambda < 1.01$), where relative errors for the expected values range between 5% and 10%. This behavior is consistent with the approximation limitations observed in the PCE model at the filament level.

To characterize how the stochastic variability of the microstructural parameters propagates to the macroscopic scale, we analyzed the CoV of the homogenized stress response. A consistent trend of uncertainty amplification was observed across all deformation modes. In the small deformation regime ($\gamma \approx 0.1$ and $\lambda \approx 1.05$), the output CoV remains moderate, generally around 35%. However, as the deformation increases, the CoV rises sharply to approximately 48-53%. Under simple shear, the shear stress CoV increases from 35.8% at $\gamma =0.1$ to 48.5% at $\gamma =0.25$. A similar tendency is observed in uniaxial and biaxial tension, where the normal stress CoV reaches 51.3% and 53.1%, respectively, at maximum stretch. As the filaments extend and stiffen, small variations in microstructural parameters produce magnified fluctuations in the resulting stress. Notably, this amplification is independent of the macroscopic deformation mode, suggesting that uncertainty propagation is governed primarily by local filament stretch distributions. Furthermore, in simple shear, the induced normal stress exhibits a CoV of 51.2%, slightly higher than that of the shear stress (48.5%). This indicates that second-order effects are similarly sensitive to material uncertainty.

Regarding efficiency, we analyzed the cost at the filament level. The original mechanical model involves calculating the filament force using an iterative implicit scheme (Brent’s method), which requires $\approx 2\times 10^{-6} {~s}$ per call (AMD EPYC^TM^ 7513 processor). In contrast, the evaluation of a polynomial consists of explicit algebraic operations with negligible cost on the order of nanoseconds. This filament-level difference can become significant when projected to higher-scale frameworks. The cumulative effort of the original model scales with the number of evaluations: summing over the discrete filament directions per integration point, across the entire finite element mesh. Furthermore, achieving a statistical description comparable to the surrogate would require implicitly solving the deterministic equations for a large number of samples. This entails either averaging over multiple material configurations for each filament orientation, or performing $\approx {1000}$ MCS of the FE model.

### Bulk rheology

The bulk rheology setup demonstrates that the Network Surrogate reproduces the statistical response of the material in larger, more complex models, involving a higher number of elements. Results are benchmarked against 10 000 MCS of the original network model. For this analysis, only three distinct PCE models were constructed, one for each quantity of interest, and evaluated uniformly across all mesh elements and deformation increments. While this setup assumes a statistically homogeneous microstructure, the proposed framework can be readily extended by mapping PCEs derived from different input distributions, thereby capturing the influence of crosslinker concentrations that vary spatially or evolve with deformation.

**Fig. 11:**
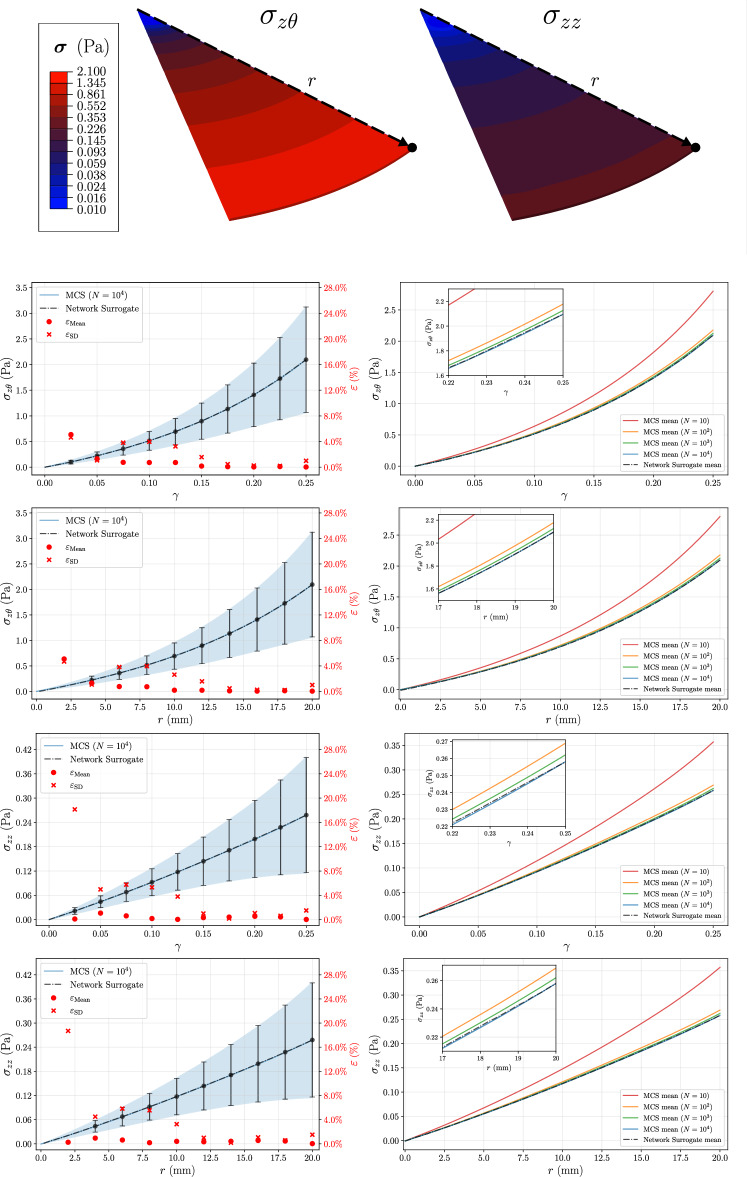
Bulk rheology simulation results. Top: spatial distributions of shear and normal stresses (log-scale color bar). Remaining panels show quantitative comparisons across radial positions on the gel surface and over the deformation history at the marked node (black dot). Left column compares mean and standard deviation of reference MCS ($N=10^4$) and Network Surrogate, with red markers indicating absolute percentage error at each point. Right column displays convergence of the MCS mean for increasing sample sizes alongside the Surrogate mean

In order to confirm that the surrogate captures not only the deformation history but also spatial variations, Fig. 11 presents results for both shear and normal stresses at a node located on the outer edge of the gel layer over the full deformation history, as well as for increasing radial position at maximum shear strain ($\mathrm \gamma =0.25$).

Both expected values and standard deviation curves from the Network Surrogate closely match the results from 10 000 MCS. For the expected value, the filament surrogate produces a closer estimate to the reference response than MCS with $N=10^3$ samples, with the increasing sample size converging toward the surrogate predictions. The absolute percentage errors displayed for the loading history and radial distribution further confirm the good performance of the proposed methodology, with the highest relative errors for both statistical moments being observed for lower strain levels, as the PCE evaluations have shown to be less accurate in this deformation regime. Nonetheless, for the lowest strains, errors remain below 5% for the expected values and below 19% for the standard deviation, which represents stress differences on the order of $10^{-3}$ Pa and $10^{-2}$ Pa, respectively. Overall, the surrogate predicts mean responses more accurately than variability. Across all deformation levels and radial positions considered, except the lowest point for each, the predicted mean normal and shear stresses have relative errors very close to or below 1%. For the standard deviation, lower deformation values and radial positions exhibit a $\varepsilon _{\text {SD}}$ ranging between 4% and 8%, before decreasing to near 1% in the mid-upper range. For the maximum shear strain, this value slightly increases, while maintaining a good accuracy with $\varepsilon _{\text {SD}}<2\%$, thus showing that the model marginally underestimates the network variability in these conditions.

The high accuracy of the macroscopic moments confirms the polynomial degree $p=5$ as a suitable truncation for this framework. As the main objective is robust uncertainty quantification, the current surrogate meets this requirement by recovering the first and second statistical moments with relative errors below 1% and 5%, respectively, across the bulk of the deformation range. Although extending the polynomial degree to $p=6$ could yield marginal improvements in filament-level predictions, such refinement is expected to yield limited gains in the accuracy of the macroscopic moments. Hence, the 5^th^-order discretization adequately captures the relevant stochastic physics of the network, suggesting that further complexity is unnecessary.

The stress distributions further illustrate that the shear stress reaches a highly nonlinear behavior, while the normal stress response varies almost linearly throughout the deformation path and radial position. Moreover, the ratio between normal and shear stress is substantially larger than in the simple shear deformation case for the unit cube, suggesting the filaments become more aligned in the shear direction under the current boundary conditions, with a significantly smaller contribution to the normal stress.

**Fig. 12:**
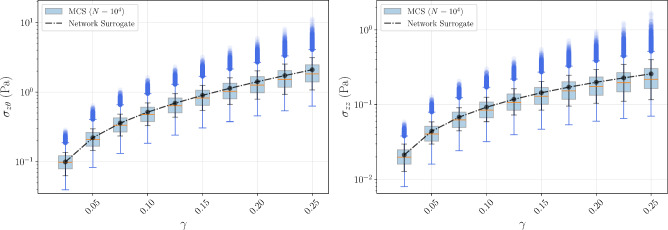
Mean and standard deviation for normal and shear stresses for the Network Surrogate against boxplots for their distribution in 10 000 MCS

**Fig. 13:**
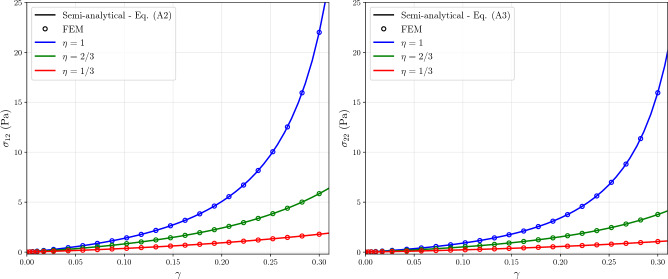
Verification of the finite element implementation against semi-analytical solutions for simple shear deformation. The numerical results (circles) are compared with the analytical solution (solid lines) for both shear ($\sigma _{12}$) and normal ($\sigma _{22}$) stress components. Different colors correspond to varying values of the crosslinker relative stiffness ($\eta$)

In addition, normal stress evolution exhibits a plateau in the lower region of the variation band. To further investigate this behavior, Fig. 12 compares the expected value from the Network Surrogate’s predicted normal and shear stresses against the quantile distribution of the full MCS set, for each deformation increment for a node on the gel’s outer edge, identifying the outliers in each distribution. There is a marked increase in the number of outliers with increasing deformation. This effect is more pronounced in the normal stress, where the number of outliers starts at 284 for the minimum deformation value, reaching 584 at maximum deformation (5.8% of the full set). These extreme responses likely correspond to samples where the filament end-to-end distance has reached or is very close to the contour length, possibly combined with a greater crosslinker relative stiffness. The accumulation of high-stress outliers skews the distributions toward larger values, which calls for additional descriptors of the filament response, since the first two moments alone might not adequately characterize the full distribution. To better represent such skewed behavior, the PCE surrogate could be augmented with models that directly target distribution tails and quantiles, for example, quantile-regression-based machine learning approaches (Koenker and Bassett 1978). Such a hybrid approach would improve characterization of extreme responses and increase the surrogate’s utility in clinically relevant applications.

Nevertheless, the current Network Surrogate already provides a highly efficient and accurate tool for estimating the mechanical response of F-actin networks. By capturing the variability due to uncertainty in filament material parameters, it reduces reliance on extensive MCS. Being implemented at the filament level, the surrogate removes the need to modify the equilibrium equations of the full network model, something that intrusive stochastic approaches would require, while still delivering accurate estimates of higher-order behavior. Finally, the high scalability of the proposed method - where the required dataset can be generated directly by solving the implicit Eqs.  (4), (5) and (8), constitutes a clear advantage over surrogates trained on FEM simulations. Such FEM-level surrogates require substantially greater computational resources to train and are typically tied to specific geometries and boundary conditions. In addition, they commonly report results only for a single node or a small region, rather than delivering element-level, full-field predictions.

## Conclusions

In this work, the PCE method was adopted to build surrogate models in the context of crosslinked F-actin filaments, focusing on three quantities of interest: filament force *f* and the first and second derivatives of the free energy with respect to the filament stretch, $w^\prime$ and $w^{\prime \prime }$, respectively. This surrogate model was then integrated at the network level, allowing for the expansion of the uncertainty in the response of a higher scale model.

PCE evaluation showed that the surrogate demonstrates strong overall accuracy in predicting the filament quantities of interest, despite some limitations in the accuracy of predictions for near-zero *f* and $w^{\prime }$ values. A sensitivity analysis revealed that the contour length *L* and filament end-to-end distance $r_{0,f}$ are the most influential parameters in the filament response, while the crosslinker end-to-end distance $r_{0,c}$ and stretch modulus $\mu _0$ have a negligible effect for the range of parameters considered.

By comparing the Network Surrogate results with those obtained from MCS for three loading cases (simple shear, uniaxial and biaxial), it was shown that the Network Surrogate is a computationally efficient and accurate approach to model the uncertainty in the mechanical response of crosslinked F-actin networks, as it is able to capture the statistical behavior of a high number of realizations. The Network Surrogate captured the expected value of the stress response at maximum deformation with an error below 1% when compared to the reference MCS with $N=10^4$ samples, while also presenting better predictions than MCS with $N=10^3$ samples.

A FEM example of a rheological setup was provided, validating the proposed framework in a more complex boundary value problem. The Network Surrogate was able to accurately predict the expected value and standard deviation of both normal and shear stresses, with errors close to 1% for the greater part of the loading history. The stress distributions revealed a highly nonlinear behavior for the shear stress, while the normal stress response varied almost linearly across the deformation path and radial coordinate.

As a proof of concept demonstrating the effectiveness of using PCEs to model uncertainty in F-actin networks, this framework suggests several pathways for future development. First, increasing the stretch regime of the filament model could enable more extensive exploration of the filament and network behavior under large deformations. However, due to the extreme variations in the quantities of interest over such increased stretch ranges, it is likely that "local" PCEs (i.e., different PCEs for different stretch intervals) would be required to maintain accuracy. Additionally, the description of the network behavior could be complemented by the use of machine learning models based on quantile regression, to better capture the tails of the stress distributions observed in the FEM example. Finally, incorporating microstructure-informed viscoelastic material behavior, with the diffusion of crosslinkers and transient binding/unbinding dynamics governing the dynamic evolution of the network architecture, would give rise to a new framework for in silico studies.

## Data Availability

The data that support the findings of this study are available from the corresponding author upon reasonable request.

## FE Model Validation

The numerical implementation was verified using a single-element unit cube (C3D8) under simple shear, characterized by the deformation gradient:

A1 $$\begin{aligned} \textbf{F} = \begin{bmatrix} 1 & \gamma & 0 \\ 0 & 1 & 0 \\ 0 & 0 & 1 \end{bmatrix} ~\mathrm {.} \end{aligned}$$

The validation relies on the semi-analytical framework presented by Holzapfel et al. (2014), which includes a detailed discussion on the appropriate integration limits. Following this formulation, the shear stress component $\sigma _{12}$ for the affine network is given by:

A2 $$\begin{aligned} \sigma _{12} \!=\! n \int _{\Omega } \lambda ^{-1} w^{\prime }(\lambda ) \sin \Phi (\cos \Phi \!+\! \gamma \sin \Phi ) \sin ^3 \Theta \, d\Theta \, d\Phi \nonumber \\ \end{aligned}$$

while the normal stress component $\sigma _{22}$ is defined as:

A3 $$\begin{aligned} \sigma _{22} \!=\! n \int _{\Omega } \lambda ^{-1} w^{\prime }(\lambda ) \left( \sin ^2 \Phi \sin ^3 \Theta \!-\! \cos ^2 \Theta \sin \Theta \right) d \Theta d \Phi \nonumber \\ \end{aligned}$$

The first derivative of the free energy is substituted according to Eq. (5), employing the extensible filament model with compliant crosslinkers defined in Eq. (4). Figure 13 presents the comparison between the numerical and analytical results. The excellent agreement between FEM predictions and semi-analytical solutions validates both the stress update algorithm and the corresponding tangent stiffness matrix.
